# Effect of medication adherence on disease activity among Japanese patients with rheumatoid arthritis

**DOI:** 10.1371/journal.pone.0206943

**Published:** 2018-11-02

**Authors:** Shunsaku Nakagawa, Mayumi Nakaishi, Motomu Hashimoto, Hiromu Ito, Wataru Yamamoto, Ran Nakashima, Masao Tanaka, Takao Fujii, Tomohiro Omura, Satoshi Imai, Takayuki Nakagawa, Atsushi Yonezawa, Hirohisa Imai, Tsuneyo Mimori, Kazuo Matsubara

**Affiliations:** 1 Department of Clinical Pharmacology and Therapeutics, Kyoto University Hospital, Kyoto, Japan; 2 Department of Advanced Medicine for Rheumatic Diseases, Graduate School of Medicine, Kyoto University, Kyoto, Japan; 3 Department of Orthopedic Surgery, Graduate School of Medicine, Kyoto University, Kyoto, Japan; 4 Department of Health Information Management, Kurashiki Sweet Hospital, Kurashiki, Japan; 5 Department of Rheumatology and Clinical Immunology, Graduate School of Medicine, Kyoto University, Kyoto, Japan; 6 Department of Clinical Immunology and Rheumatology, Wakayama Medical University, Wakayama, Japan; 7 Graduate School of Medicine, Department of Medical and Pharmaceutical Community Healthcare, the University of Tokyo, Tokyo, Japan; Keio University, JAPAN

## Abstract

For the optimum efficacy of disease-modifying anti-rheumatic drugs (DMARDs), patients need to be adherent to their medication regimen. To clarify the effects of medication adherence on disease activity in Japanese patients with rheumatoid arthritis (RA), we conducted a cohort study in patients with various stages of RA. Patients were enrolled from the Kyoto University RA Management Alliance cohort, and followed up prospectively for 12 months. In this study, a total of 475 patients were analyzed and divided into 9 groups according to their medication adherence and the RA disease duration. The primary outcomes were based on the rate of a disease flare. The secondary outcomes were the changes in disease activity score using 28 joints (DAS28-ESR), simplified disease activity index (SDAI) and physical disability by health assessment questionnaire-disability index (HAQ). The changes in DAS28-ESR, HAQ, and the risk of disease flare in the highly adherent patients were significantly lower than those of the less adherent patients among the groups with RA ≤ 4.6 years but not those among the other groups. Taken together, this study identified a significant association between medication adherence and the disease flare during early-stage RA or short disease duration. These results emphasize the need to pay more attention to medication adherence in preventing the disease progression of RA.

## Introduction

Rheumatoid arthritis (RA) is a chronic inflammatory disease that can result in severe disability and morbidity. Highly effective treatment with disease-modifying anti-rheumatic drugs (DMARDs) has been shown to have a major role in improving clinical outcomes of RA patients. However, the optimum efficacy of these drugs needs patients to be fully adherent to their medication regimen. Medication adherence is defined by the World Health Organization (WHO) as an extent to which a person’s behavior (e.g. taking medications according to instruction, following dietary guidance, and/or executing lifestyle changes recommended by healthcare providers [1]. In clinical situations, patients’ adherence can be assessed by direct methods (such as pill counts or measurement of plasma-drug concentrations), and by indirect methods (such as prescriptions records or self-reported questionnaires). A systematic literature review has revealed that 66% of all patients actually adherent to their treatment regimen for RA, and differences in methods to assess patient adherence did not significantly affect the results [2]. Medication adherence of RA patients is affected by age, sex, ethnicity, type of medication, disease duration [2–5]. In addition, it has been reported that rates of adherent patients are relatively higher among patients with acute-phase RA as compared with those with chronic-phase RA [6, 7].

Several studies have demonstrated an association between higher medication adherence and better clinical response to therapies in RA patients [8–13]. Of these investigations, one has indicated that medication adherence is associated with improvements in disease activity and physical functional outcomes among DMARDs-naïve patients, but not among existing users [13]. These findings suggest that medication adherence is a significant factor in treatment of early-stage RA. Similarly, low adherence has been previously reported to be associated with flares in disease activity of RA [14]. Although this result was obtained from a small sample-size, it has been suggested that RA patients who took their medications irregularly might have fluctuations in their disease activity and show more rapid progression in the disease. Further studies are needed to verify this hypothesis.

Based on these findings, we endeavored to clarify the effects of medication adherence on disease activity of Japanese patients with RA. For this purpose, we conducted a cohort study in patients with various stages of RA.

## Materials and methods

### Setting and study population

A total of 563 RA patients were enrolled from the KURAMA (Kyoto University Rheumatoid Arthritis Management Alliance) cohort and followed up prospectively. The KURAMA cohort was established in 2011 at the Center for Rheumatic Diseases in Kyoto University Hospital for tight/proper control of RA and utilization of their sequential clinical and laboratory data for clinical investigations as described in detail previously [15–17]. In the current study, we enrolled all the patients consecutively from 1st May to 31st December, 2015. All patients fulfilled the revised 1987 American College of Rheumatology (ACR) criteria for RA or the 2010 ACR/ European League Against Rheumatism (EULAR) classification criteria for RA and provided written informed consent. This study was designed in accordance with the Declaration of Helsinki and was approved by the Medical Ethics Committee of Kyoto University Graduate School and Faculty of Medicine before starting the study (R0357). In this study, there was no formal sample size calculation as we used all available data from the KURAMA cohort. We followed up all the inclusion patients for 12 months.

All patients who answered the questionnaire related to medication adherence during the study period were included (n = 563). From a total of 563 patients, the patient who did not fully answer the questionnaire on medication adherence (n = 1), patients who did not use any medications for RA at baseline (n = 8), patients without data on the RA disease duration (n = 8), patients without data on the RA disease activity at baseline (n = 33), and patients without data on the RA disease activity during follow-up period (n = 38) were excluded from the subsequent analysis.

### Evaluation of medication adherence

We referred to previous studies on medication adherence of patients with chronic disease [18–20], and designed a 6-item questionnaire related to priority for medication, concern about side effects or burden of taking the prescribed medication. The questionnaire consists of the following five yes/no questions: about patient’s tendency 1) “Do you sometimes miss-dosing your medications?”, 2) “When you go out or travel, does it sometimes happen that you forget to carry your medications?”, 3) “Over the last 2 weeks, were there any days you did not take your medicines?”, 4) “When you feel like your symptoms or disease state are under control, do you sometimes stop taking your medications?”, and 5) “Have you ever cut down or stopped taking your medicines before telling your doctor because you felt worse when you took them?” The responses were graded either 0 (for ‘Yes’) or 1 (for ‘No’), accordingly. Question 6 was: “How often do you think that it is difficult to take all your medicines?” which was graded as 1 (all the time) to 5 (never). This questionnaire was validated in independent 61 RA patients (disease duration, 8.5 ± 8.4 years). The Cronbach’s alpha coefficient for this questionnaire was calculated as 0.75 to ensure internal consistency. Then, these 6 items related to medication adherence were processed by exploratory factor analysis using the principal factor method. The factor loading for each item was calculated with range 0.15–0.76 (for #1, 0.75; for #2, 0.68; for #3, 0.15; for #4, 0.68; for #5, 0.59; for #6, 0.76). Since the Bartlett’s test of sphericity was significant (chi-square, 119.7; p < 0.0001), a sum of scores derived from the 6-item questionnaire was used to evaluate medication adherence of each patient. The sum of scores ranged from 0 to 10. Patients were considered to have high, moderate or low medication adherence if the sum of scores was 10, 8–9 or 1–7, respectively. For 475 patients in the subsequent analysis, the Cronbach’s alpha coefficient for this questionnaire was calculated as 0.71.

### Data collection and evaluation of disease activity

Clinical characteristics evaluated included age, sex, disease duration of RA, RA stage defined by Steinbrocker classification [21], use of medication for RA, doses for methotrexate (MTX) and prednisolone, experience with adverse drug reactions, hospital admission within the last 12 months, presence/absence of extra articular disease, prior tobacco use, and baseline values of aspartate aminotransferase (AST), alanine aminotransferase (ALT), lactate dehydrogenase (LDH), triglyceride (TG), hemoglobin A1c (HbA1c), estimated glomerular filtration rate (eGFR), blood urea nitrogen (BUN) and C-reactive protein (CRP). Adalimumab, certolizumab, infliximab, tocilizumab, abatacept, etanercept and tofacitinib were categorized as biological DMARDs (bioDMARDs). Actarit, aurothiomalate, auranofin, bucillamine, iguratimod, leflunomide, mizoribine, salazosulfapyridin, cyclosporine and tacrolimus were categorized as conventional synthetic DMARDs (csDMARDs). Disease activity of RA was evaluated by disease activity score using 28 joints (DAS28-ESR), simplified disease activity index (SDAI), physical disability by health assessment questionnaire-disability index (HAQ). The DAS28-ESR disease activity of RA was defined as follow: DAS28-ESR <2.6, remission; DAS28-ESR <3.2 for low disease activity; DAS28-ESR ≤5.1 for moderate disease activity; and DAS28-ESR >5.1 for high disease activity. Baseline was defined as the date when the medication adherence was measured. All data observed during 12 months from baseline was collected.

### Statistical analysis

The primary outcomes included the rate of a disease flare [22]. Disease flare was defined as an increase in DAS28-ESR >0.6 and DAS28-ESR at endpoint >3.2. An increase in DAS28-ESR was calculated as a difference between the maximum DAS28-ESR during the follow-up period and the minimum value of previously observed DAS28-ESR. The Kaplan-Meier survival method was used to visually evaluate the relationship between medication adherence and outcomes, with statistical comparison using the log-rank test. We used the difference between the date when medication adherence was initially evaluated and the date of final assessment as the censored time. The effects of medication adherence on the time to disease flare were expressed as hazard ratios with 95% confidence intervals, estimated by Cox regression adjusted to a linear term of the propensity score [23]. For each patient, we calculated the propensity score, defined as the conditional probability of a patient being highly adherent at baseline given confounders, age, sex, disease duration of RA, RA class, RA stage, medication for RA, hospital admission in the last 12 months, presence/absence of extra articular disease, smoking, baseline DAS28-ESR, baseline SDAI, baseline HAQ, and the baseline values of laboratory date (Hemoglobin, White blood cell count, AST, ALT, TG, HbA1c, eGFR and BUN) using regression and single mean imputation for missing covariates. The proportional hazards assumption was confirmed with log-negative log graphs.

The secondary outcomes were the changes in RA disease activity. Disease progression was assessed by the increase in DAS28-ESR, SDAI or HAQ. An increase in disease activity index was calculated as a difference between the maximum value during the follow-up period and the minimum of previously observed value. The effects of medication adherence were estimated by comparing the changes in disease activity between the highly adherent and less adherent patients. As described in detail previously [13, 24], we determined adjusted estimates using inverse propensity score weighted (IPSW) generalized estimating equations modeled with the linear link function. The entire study-sample was based on the inverse of the propensity score where the weight for the highly adherent patient was equal to the inverse of the propensity score, while the weight for the patient with moderate or low adherence was equal to the inverse of 1 minus the propensity scores.

Data were expressed as the mean ± standard deviation for continuous variables, and numbers (%) for categorical variables. Continuous variables were assessed by the Jonckheere-Terpstra test. The Cochran-Armitage test was used to compare categorical variables. To find clusters of variables, we used a principal component analysis. All reported probability values were two-sided, and we considered P < 0.05 to be statistically significant. All analysis was done using JMP software (version 12.0) or EZR (Saitama Medical Center, Jichi Medical University, Saitama, Japan), which is a graphical user interface for R (The R Foundation for Statistical Computing, Vienna, Austria) [25].

## Results

### Baseline demographics and clinical characteristics in study population

The included patients (n = 475) were subjected to the following analysis. The baseline demographics and clinical characteristics are summarized in Table 1. Based on the self-reported questionnaire on medication-taking behavior, patients were divided into 3 groups: the patients with high, moderate or low adherence. Based on a previous study that medication adherence had marked influence in new DMARDs users [13], we assumed that medication adherence had greater influences in patients with early RA stage compared to those with RA later-stage. In this study, we could not obtain data on DMARDs naïve patients; however, disease duration would be correlated with the treatment duration. Thus, the study population was further divided into 3 groups based on disease duration (158 patients with RA ≤ 4.6 years, 158 patients with RA 4.7–13.6 years, 159 patients with RA ≥ 13.7 years). In patients with disease duration ≤ 4.6 years, the medication adherence were significantly associated with age, RA stage, AST, eGFR and BUN. Among the patients with 4.7–13.6 years of disease duration, the medication adherence were significantly associated with age, prednisolone use, AST, HbA1c, eGFR and BUN and the rate of hospital admission within the last 12 months. In patients with RA ≥ 13.7 years, the medication adherence were significantly associated with baseline DAS28-ESR and baseline SDAI. A principal component analysis showed 2 clusters of these variables: the first cluster included age, eGFR, BUN, AST, HbA1c and the rate of hospital admission within the last 12 months; and the second cluster included a baseline DAS28-ESR, baseline SDAI, RA stage and prednisolone use.

**Table 1:** Baseline demographics of study population with rheumatoid arthritis (RA).

Disease duration	≤ 4.6 years			4.7–13.6 years			≥ 13.7 years		
Medication adherence	High	Moderate	Low	High	Moderate	Low	High	Moderate	Low
Number of patients	80	42	36	76	47	35	88	49	22
Mean adherence score	8.6 ± 1.8			8.7 ± 1.8			9.1 ± 1.4		
Age, year	63.5 ± 12.2*	61.0 ± 15.0	55.0 ± 16.9	66.5 ± 12.1**	59.3 ± 11.9	51.0 ± 14.8	68.4 ± 9.2	65.7 ± 12.2	66.5 ± 10.5
Disease duration, year^†^	3.1 ± 1.0	2.9 ± 1.0	2.9 ± 1.1	8.5 ± 2.5	7.9 ± 2.6	8.0 ± 2.6	26.7 ± 11.2	23.9 ± 8.2	25.3 ± 9.4
Sex, n (Male/Female)	21/59	14/28	12/24	17/59	3/44	7/28	9/79	6/43	2/20
RA Stage, n (1/2/3/4)	38/29/11/2*	21/15/5/1	26/7/3/0	12/27/22/15	15/17/5/10	13/12/4/6	2/8/20/58	3/2/12/32	0/2/8/12
DAS28-ESR	2.5 ± 1.0	2.4 ± 0.8	2.2 ± 0.9	2.8 ± 1.1	2.5 ± 1.0	2.4 ± 0.8	3.2 ± 1.1*	3.0 ± 1.0	2.8 ± 0.8
DAS28-ESR disease activity, n (remission/low/moderate/high)	50/12/16/2	29/6/7/0	28/5/3/0	38/13/23/2	30/8/8/1	23/5/7/0	30/16/37/5	22/10/14/3	10/6/6/0
SDAI^†^	4.2 ± 4.8	3.4 ± 3.3	4.0 ± 5.2	5.7 ± 5.1	5.0 ± 4.5	4.5 ± 4.2	7.7 ± 5.5*	6.4 ± 5.1	5.2 ± 4.1
HAQ	0.38 ± 0.51	0.39 ± 0.46	0.37 ± 0.54	0.65 ± 0.75	0.54 ± 0.70	0.35 ± 0.40	1.00 ± 0.83	0.81 ± 0.69	0.82 ± 0.70
CRP^†^	0.4 ± 1.0	0.2 ± 0.3	0.2 ± 0.3	0.6 ± 1.2	0.4 ± 0.8	0.2 ± 0.3	0.5 ± 0.9	0.6 ± 1.1	0.4 ± 0.6
Extra articular disease, n (%)	77 (96.3)	37 (88.1)	36 (100)	67 (88.2)	45 (95.7)	32 (91.4)	69 (78.4)	39 (79.6)	16 (72.7)
Pulmonary injury, n (%)	69 (86.3)	33 (78.6)	30 (83.3)	58 (76.3)	40 (85.1)	28 (80.0)	65 (73.9)	35 (71.4)	13 (59.1)
MTX use, n (%)	64 (80.0)	31 (73.8)	27 (75.0)	49 (64.5)	31 (66.0)	25 (71.4)	54 (61.4)	36 (73.5)	12 (54.6)
Dose of MTX, mg/week (mean of users)	7.7 ± 2.8	7.6 ± 2.1	8.2 ± 3.2	7.9 ± 4.1	7.2 ± 3.1	7.7 ± 3.4	6.5 ± 2.8	7.3 ± 2.8	6.3 ± 3.3
bioDMARDs use, n (%)	39 (48.8)	19 (45.2)	17 (47.2)	35 (46.1)	22 (46.8)	19 (54.3)	44 (50.0)	21 (42.9)	9 (40.9)
csDMARDs use, n (%)	25 (31.3)	15 (35.7)	18 (50.0)	25 (32.9)	20 (42.6)	11 (31.4)	34 (38.6)	20 (40.8)	11 (50.0)
Prednisolone use, n (%)	8 (10.0)	8 (19.1)	4 (11.1)	24 (31.6)*	8 (17.0)	5 (14.3)	41 (46.6)	18 (36.7)	9 (40.9)
Dose of Prednisolone, mg/day (mean of users)	3.4 ± 1.5	4.3 ± 3.3	3.0 ± 1.4	3.8 ± 2.3	2.4 ± 1.4	4.8 ± 2.6	3.9 ± 1.7	4.8 ± 4.2	3.8 ± 1.8
Hemoglobin, g/dL^†^	12.9 ± 1.3	13.1 ± 1.4	12.7 ± 1.5	12.6 ± 1.7	12.4 ± 1.5	12.8 ± 1.7	12.4 ± 1.4	12.4 ± 1.6	13.0 ± 1.2
White blood cell, 10^9^ cells/L^†^	5.52 ± 1.70	5.62 ± 1.47	5.59 ± 1.90	6.13 ± 2.28	5.39 ± 1.64	5.77 ± 1.85	6.37 ± 2.00	6.31 ± 2.51	6.89 ± 2.01
AST, U/L^†^	26 ± 10*	23 ± 6	27 ± 31	26 ± 12*	25 ± 11	22 ± 7	23 ± 6	26 ± 11	25 ± 8
ALT, U/L^†^	23 ± 13	21 ± 10	31 ± 67	22 ± 17	21 ± 14	19 ± 12	16 ± 7	21 ± 19	18 ± 9
TG, mg/dL^†^	137 ± 122	119 ± 64	124 ± 94	120 ± 78	101 ± 61	114 ± 63	112 ± 53	108 ± 54	127 ± 65
HbA1c, %^†^	5.4 ± 0.6	5.2 ± 0.4	5.2 ± 0.3	5.3 ± 0.4**	5.2 ± 0.3	5.1 ± 0.3	5.3 ± 0.4	5.2 ± 0.4	5.4 ± 0.4
eGFR, mL/min/1.73m^2^^†^	71.6 ± 17.5**	76.2 ± 14.9	80.6 ± 15.9	72.2 ± 18.2**	75.6 ± 15.7	84.1 ± 21.5	74.9 ± 23.9	73.3 ± 18.3	72.8 ± 22.5
BUN, mg/dL^†^	16 ± 5**	15 ± 4	13 ± 4	16 ± 5**	15 ± 4	14 ± 4	17 ± 6	16 ± 4	17 ± 5
Experience with adverse drug reaction, n (%)^†^	54 (67.5)	27 (64.3)	23 (65.7)	34 (45.3)	19 (40.4)	16 (45.7)	34 (38.6)	25 (51.0)	6 (27.3)
Hospital admission in the last 12 months, n (%)	10 (12.5)	9 (21.4)	5 (13.9)	19 (25.0)*	5 (10.6)	3 (8.6)	19 (21.6)	4 (8.2)	4 (18.2)
Ever smoking, n (%)^†^	12 (15.0)	6 (14.3)	10 (28.6)	8 (10.7)	6 (12.8)	6 (17.1)	6 (6.8)	3 (6.1)	1 (4.6)

RA stage, Steinbrocker classification; DAS28, disease activity score using 28 joints; SDAI, simplified disease activity index; HAQ, health assessment questionnaire-disability index; CRP, C-reactive protein; MTX, methotrexate; AST, aspartate aminotransferase; ALT, alanine aminotransferase; TG, triglyceride; eGFR, estimated glomerular filtration rate; BUN, blood urea nitrogen. bioDMARDs includes adalimumab, certolizumab, infliximab, tocilizumab, abatacept, etanercept and tofacitinib. csDMARDs includes actarit, aurothiomalate, auranofin, bucillamine, iguratimod, leflunomide, mizoribine, salazosulfapyridin, cyclosporine and tacrolimus. Adherent and non-adherent patients were compared at different stages of RA. The DAS28-ESR disease activity of RA was defined as follows: DAS28-ESR < 2.6, remission; DAS28-ESR < 3.2, low disease activity; DAS28-ESR ≤ 5.1, moderate disease activity; DAS28-ESR > 5.1, high disease activity. Data were expressed as the mean ± standard deviation for continuous variables, and numbers (%) for categorical variables. Continuous variables were assessed by the Jonckheere-Terpstra test. The Cochran-Armitage test was used to compare categorical variables. The results of these test were shown with symbols if the p values for trend were < 0.05 (*) or P < 0.01 (**).

^†^Missing data: 1 in SDAI, 2 in experience with adverse drug reaction and smoking history; 19 in HbA1c; 20 in CRP and TG; 21 in hemoglobin and white blood cell count; 25 in AST, eGFR and BUN; 26 in ALT.

### Effects of medication adherence on disease flare

**Fig 1:**
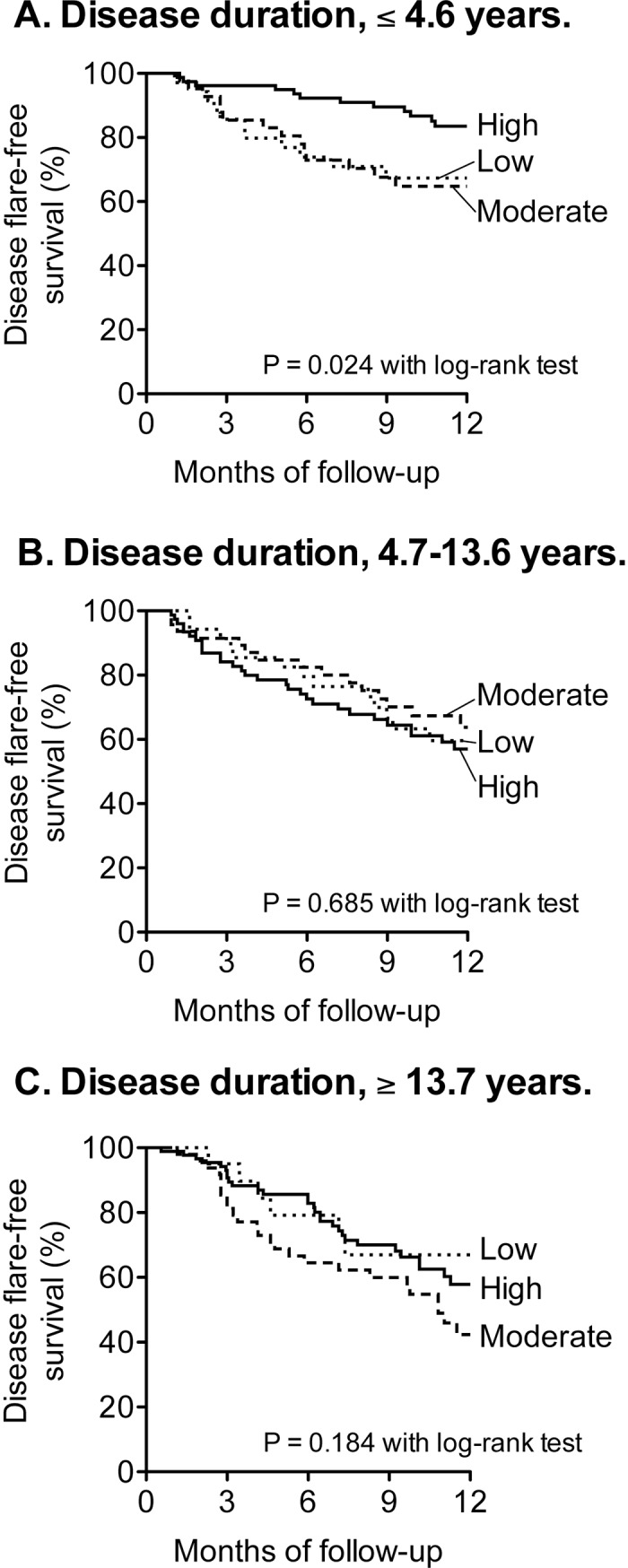
The association between medication adherence and disease flare. Patients (%) with disease flare were examined. The study population was divided into 3 groups based on disease duration: (A) 158 patients with RA ≤ 4.6 years, (B) 158 patients with RA 4.7–13.6 years, (C) 159 patients with RA ≥ 13.7 years. Disease flare was defined as an increase in DAS28-ESR > 0.6 and DAS28-ESR at endpoint > 3.2. The Kaplan-Meier survival method was used to visually evaluate the relationship between medication adherence and the outcomes, with statistical comparison using the log-rank test.

The relationship between medication adherence and the flare-free disease survival is shown in Fig 1. The proportions of disease flare in highly adherent patients with RA ≤ 4.6 years were remarkably lower than those of other patients. The effects of medication adherence on the time to disease flare were expressed as hazard ratios with 95% confidence intervals, and estimated by Cox regression (Table 2). Among the groups with shorter disease duration (≤ 4.6 years), the crude risk of disease flare of the highly adherent patients was significantly lower than that of the patients with moderate or low adherence. This effect of medication adherence on disease flare was not attenuated after the adjustment with propensity score. However, no significant effect of medication adherence on the disease flare was observed among the patients with longer disease duration (> 4.6 years).

**Table 2:** Effects of medication adherence on the disease flare.

Subgroup	High adherence, n (with flare/total)	Moderate adherence, n (with flare/total)	Low adherence, n (with flare/total)
		Hazard ratio (95% CI)	Hazard ratio (95% CI)
All patients	70/244	54/138	30/93
Hazard ratio	Reference	1.41 (0.99 to 2.01)	1.17 (0.76 to 1.78)
Adjusted hazard ratio	Reference	1.54 (1.06 to 2.21)*	1.72 (1.06 to 2.71)*
Disease duration, ≤ 4.6 years	12/80	14/42	11/36
Hazard ratio	Reference	2.61 (1.20 to 5.74)*	2.49 (1.08 to 5.70)*
Adjusted hazard ratio	Reference	2.68 (1.22 to 5.94)*	3.25 (1.29 to 8.04)*
Disease duration, 4.7–13.6 years	29/76	15/47	13/35
Hazard ratio	Reference	0.76 (0.40 to 1.40)	0.88 (0.44 to 1.66)
Adjusted hazard ratio	Reference	1.08 (0.54 to 2.09)	1.75 (0.81 to 3.60)
Disease duration, ≥ 13.7 years	29/88	25/49	6/22
Hazard ratio	Reference	1.55 (0.90 to 2.65)	0.87 (0.33 to 1.95)
Adjusted hazard ratio	Reference	1.50 (0.86 to 2.60)	0.96 (0.36 to 2.21)
MTX user	40/167	41/98	22/64
Hazard ratio	Reference	1.85 (1.20 to 2.87)**	1.55 (0.90 to 2.58)
Adjusted hazard ratio	Reference	1.90 (1.21 to 2.97)**	2.44 (1.36 to 4.24)**
bioDMARDs user	37/118	27/62	17/45
Hazard ratio	Reference	1.50 (0.91 to 2.46)	1.30 (0.71 to 2.27)
Adjusted hazard ratio	Reference	1.62 (0.96 to 2.70)	2.05 (1.04 to 3.87)*
csDMARDs user	32/84	22/55	14/40
Hazard ratio	Reference	1.06 (0.61 to 1.82)	0.92 (0.47 to 1.69)
Adjusted hazard ratio	Reference	1.05 (0.58 to 1.87)	1.21 (0.59 to 2.36)
Prednisolone user	32/73	21/34	9/18
Hazard ratio	Reference	1.56 (0.88 to 2.69)	1.17 (0.53 to 2.36)
Adjusted hazard ratio	Reference	1.58 (0.88 to 2.77)	1.15 (0.50 to 2.40)

The effects of medication adherence on the time to disease flare were expressed as hazard ratios with 95% confidence intervals, estimated by Cox regression adjusted a linear term of the propensity score.

*P < 0.05

**P < 0.01.

Type of medication can affect the impact of adherence on disease activity. Therefore, we further performed sub-group analysis considering the type of medication (Table 2). We estimated the hazard ratios for disease flare focusing on MTX users, bioDMARDs users, csDMARDs users and prednisolone users. The results showed that medication adherence was significantly related to the decreased risk of disease flare among the patients using MTX and bioDMARDs. However, no significant effect of medication adherence was observed in the analysis focusing csDMARDs users or prednisolone users.

### Effects of medication adherence on the changes in RA disease activity

Subsequently, the effects of medication adherence on the increase in DAS28-ESR, SDAI and HAQ were examined (Table 3). Disease progression was assessed by differences between the maximum values of the disease activity index and the minimum of previously observed values. In groups with early-stage RA, the changes in DAS28-ESR of the highly adherent patients were significantly lower than those of the less adherent patients. After the adjustment using IPSW, the significant effects of medication adherence on HAQ was also detected. However, there were no significant effects of medication adherence in the group with later-stage RA.

**Table 3:** Effects of medication adherence on changes in rheumatoid arthritis (RA) disease activity.

Disease activity index	High adherence	Moderate or low adherence	Differences (95% CI)	Adjusted (95% CI)
All patients				
DAS28-ESR	0.6 ± 0.7	0.8 ± 0.9	-0.18 (-0.33 to -0.03)*	-0.20 (-0.34 to -0.05)**
SDAI	3.7 ± 5.2	4.4 ± 6.0	-0.73 (-1.74 to 0.29)	-0.90 (-1.91 to 0.11)
HAQ	0.21 ± 0.37	0.21 ± 0.34	-0.002 (-0.066 to 0.062)	-0.054 (-0.119 to 0.010)
Disease duration, ≤ 4.6 years				
DAS28-ESR	0.5 ± 0.7	0.8 ± 1.0	-0.30 (-0.57 to -0.04)*	-0.40 (-0.66 to -0.13)**
SDAI	2.8 ± 4.7	4.0 ± 7.1	-1.19 (-3.07 to 0.70)	-1.54 (-3.40 to 0.31)
HAQ	0.17 ± 0.31	0.22 ± 0.39	-0.047 (-0.157 to 0.063)	-0.133 (-0.249 to -0.016)*
Disease duration, 4.7–13.6 years				
DAS28-ESR	0.7 ± 0.7	0.9 ± 0.8	-0.13 (-0.37 to 0.11)	-0.15 (-0.38 to 0.09)
SDAI	4.0 ± 5.2	4.6 ± 5.0	-0.56 (-2.16 to 1.03)	-0.84 (-2.38 to 0.70)
HAQ	0.26 ± 0.39	0.17 ± 0.27	0.088 (-0.018 to 0.195)	0.051 (-0.052 to 0.154)
Disease duration, ≥ 13.7 years				
DAS28-ESR	0.7 ± 0.8	0.8 ± 0.8	-0.10 (-0.36 to 0.16)	-0.04 (-0.30 to 0.22)
SDAI	4.2 ± 5.7	4.7 ± 5.8	-0.48 (-2.30 to 1.34)	-0.19 (-2.04 to 1.66)
HAQ	0.19 ± 0.40	0.24 ± 0.34	-0.048 (-0.166 to 0.069)	-0.074 (-0.190 to 0.042)

The effects of medication adherence were estimated by comparing the changes in disease activity between the highly adherent patients and the less adherent patients. We determined adjusted estimates using inverse propensity score weighted generalized estimating equations modeling with linear link function.

*P < 0.05

**P < 0.01.

## Discussion

We obtained the following major finding in the present study that medication adherence had a marked influence on disease flares in patients with early-stage RA. To our knowledge, this is the first study with the largest sample-size in assessing the effects of medication adherence and the RA disease duration on the flares in disease activity.

The present results indicate that medication adherence could serve as a predictor for sustained low RA disease activity. A similar result was obtained in a previous study, which focused RA patients with clinical remission [14]. Additionally, we observed that medication adherence was significantly associated with the sustained low activity in patients with shorter disease duration. We thought that the short disease duration (≤ 4.6 years) indicated early stage RA, which has been recognized as a critical period for management. Accumulating evidence has pointed out the importance of therapeutic intervention in early-stage RA to achieve earlier and more effective disease control and less joint damage [26–28]. Taken together, these results emphasize the need for more attention on medication adherence as a means to prevent disease progression, specifically in patients with early-stage RA or shorter disease duration.

In the subgroup analysis, significant effects of medication adherence on disease flare in MTX and bioDMARDs users, but not in csDMARDs and prednisolone users were observed. However we also found that the RA disease duration was significantly altered between the type of medication (MTX users, 11.9 ± 11.6 years; bioDMARDs users, 11.7 ± 10.8 years; csDMARDs users, 13.2 ± 12.3 years; prednisolone users, 16.8 ± 13.0 years). Therefore, the differences in effects of medication adherence on the type of medication could be due to the differences in disease duration of RA. Further studies are needed to clarify whether the effects of medication adherence were different between the type of medication for RA.

A systematic review has reported that the prior use of DMARDs and patient’s belief about the medication is strongly related with medication adherence of RA patients [4]. In this study, 2 clusters of covariates; the first including DAS28-ESR, SDAI, and prednisolone use; and the second including age, HbA1c, eGFR, BUN, AST and the rate of hospital admission, were associated with the medication adherence. The first cluster including disease activity might reflect prior use of DMARDs, and the second cluster including laboratory data might reflect the patient’s belief about the medication. Interestingly, age, HbA1c and renal function, but not RA disease activity, were associated with the adherence of patients with shorter RA disease duration. Therefore, education programs that focus on the patient’s beliefs about the necessity of medication may result in better adherence and slow disease progression during early-stage RA.

Several limitations of our study should be considered. First, the generalizability of this study is limited because most of the patients (97.0%) were recruited from a single university hospital. However, the demographics of our study population were not particularly different from those of Japanese RA patients in other studies [29, 30]. Second, we assessed medication adherence once only at baseline, which may not be representative of medication adherence during the follow-up. However, we have confirmed that the score on medication-taking behavior assessed by the questionnaire was maintained over 2 years in 70% of patients, while we need to assess long-term medication adherence and investigate the relationship between changes in adherence and clinical outcomes in future studies. Third, we assessed medication adherence of each patient by an indirect method using a self-reported questionnaire. Less adherent patients may record themselves as highly adherent. In this scenario, we could have underestimated the influence of moderate or low medication adherence on disease activity. However, the present results identified the significant influence of medication adherence on disease flare, which would not be affected by different methods of monitoring medication adherence. Fourth, as we had carried out an observational study rather than a randomized trial, it is impractical to establish causality. In addition to the possibility of unmeasured confounders, we cannot entirely exclude the potential of bias owing to missing covariates. However, the use of propensity score was considered to account for confounding by variables. Fifth, we did not obtain the data on time-dependent confounders, especially the changes in medication during 12 months. Further studies considering time-dependent changes in medication would be informative.

In conclusion, this study showed a significant association between medication adherence and the disease flare in patients with short RA disease duration. It is therefore important to enhance patients’ medication adherence for RA treatment, specifically during early stage of the disease.
